# Draft Genome Sequence of *Kocuria rhizophila* RF, a Radiation-Resistant Soil Isolate

**DOI:** 10.1128/genomeA.00095-16

**Published:** 2016-03-10

**Authors:** Jalil Fallah Mehrabadi, Amir Mirzaie, Nahid Ahangar, Arian Rahimi, Hassan Rokni-Zadeh

**Affiliations:** a The Lister Laboratory of Microbiology, Tehran, Iran; b Young Researchers and Elite Club, East Tehran Branch, Islamic Azad University, Tehran, Iran; c Department of Microbiology, Faculty of Basic and Medical Science, Islamic Azad University, Zanjan Branch, Zanjan, Iran; d Department of Virology, Pasteur Institute of Iran, Tehran, Iran; e Zanjan Pharmaceutical Biotechnology Research Center, Zanjan University of Medical Sciences, Zanjan, Iran; f Department of Medical Biotechnology and Nanotechnology, Zanjan University of Medical Sciences, Zanjan, Iran

## Abstract

*Kocuria rhizophila* RF, a soil isolate from Iran, is a radiation-resistant bacterium. Only a limited amount of genomic information for radiation-resistant bacteria is currently available. Here, we report the draft genome sequence of this bacterium, providing knowledge to aid in the discovery of the genomic basis of its resistance to radiation.

## GENOME ANNOUNCEMENT

Several bacteria are able to live under extreme environments. One such harsh condition is radiation, where only a few species can grow. The most well-known radiation-resistant bacteria are *Deinococcus radiodurans* (1, 2) and *Rubrobacter radiotoleransis* (3). However, little information is available for other radio-resistant species such as *Kocuria*. Recently, we isolated and characterized a gamma radiation-resistant bacterium from Iranian local soil and designated it *Kocuria rhizophila* RF. This strain was isolated after exposure of the soil sample to a dose of 5000 Gy gamma radiation. Here we report the draft genome sequence of this soil isolate to predict the genomic features responsible for its radiation resistance.

Genomic DNA was subjected to 101-cycle paired-end sequencing with an Illumina HiSeq 2000. Following quality control of raw data (FastQC; http://www.bioinformatics.babraham.ac.uk/projects/fastqc), reads were trimmed for *de novo* assembly by Velvet (4). Assembly of 19,838,330 reads (around 710-fold median coverage) yielded 90 contigs with an *N*_50_ of 61,655 bp. The length of the longest contig was 170,711 bp. The total assembled length was 2,778,506 bp with a G+C content of 70.2%. Automated annotation using Rapid Annotations using Subsystems Technology (RAST) (5) assigned a total of 2,331 protein-coding genes, along with 47 tRNAs.

The genome carries 22 putative genes for membrane transport; 14 virulence, disease, and defense genes; and 51 putative genes for biosynthesis of vitamins, and pigments such as pyridoxin (vitamin B_6_) and folate (vitamin B_9_). The synthesis of vitamin B_6_ has been reported for several microorganisms (6, 7). One of the proposed roles for this vitamin is oxygen quencher and antioxidant, suggesting its possible function in radiation resistance by inactivation of free toxic radicals generated upon exposure to radiation (8). Similarly, an old study showed the effect of vitamin B_9_ on resistance of *E. coli* to radiation (9). Lipoic acid and thioredoxin are two antioxidants whose biosynthetic pathways were detected in the genome of *Kocuria rhizophila* RF. Lipoic acid is a sulfuric compound used as an antioxidant in pharmaceutical industries. Likewise, thioredoxin, found in almost every microorganism, has a key role in response to reactive oxygen species (ROS). For instance, its role in resistance of alga *Spirogyra varians* to gamma radiation has been reported (10).

In addition, 13 genes can be linked to regulation and cell signaling, however, the genome lacked any homologies of a quorum-sensing regulation system.

One of the main strategies of microorganisms for resisting radiation is coping with oxidative stress by several enzymes involved in detoxification of free radicals and repair of DNA damage (11–13). For this reason, the genome of radio-resistant bacteria usually contains several oxidative stress-related genes compared to other bacteria. Accordingly, the *Kocuria rhizophila* RF draft genome carries catalase (katE), Mn catalase (katA), Alkyl hydroperoxide reductase (ahpC), dyp-type peroxidase, peptide methionine sulfoxide reductase (msrA), and Fe/Mn superoxide dismutase (soda), putatively involved in oxidative stress.

### Nucleotide sequence accession numbers.

This whole-genome shotgun project has been deposited at DDBJ/EMBL/GenBank under the accession number JPWX00000000. The version described in this paper is version JPWX02000000.
